# Astrocytic and microglial cells as the modulators of neuroinflammation in Alzheimer’s disease

**DOI:** 10.1186/s12974-022-02565-0

**Published:** 2022-08-17

**Authors:** Deepali Singh

**Affiliations:** National Brain Research Centre, Manesar, Haryana 122052 India

**Keywords:** Microglia, Astrocytes, Neuroinflammation, Amyloid beta, Alzheimer’s disease

## Abstract

Neuroinflammation is instigated by the misfiring of immune cells in the central nervous system (CNS) involving microglia and astrocytes as key cell-types. Neuroinflammation is a consequence of CNS injury, infection, toxicity, or autoimmunity. It is favorable as well as a detrimental process for neurodevelopment and associated processes. Transient activation of inflammatory response involving release of cytokines and growth factors positively affects the development and post-injury tissue. However, chronic or uncontrolled inflammatory responses may lead to various neurodegenerative diseases, including Alzheimer's disease (AD), Parkinson's disease (PD), amyotrophic lateral sclerosis, and multiple sclerosis. These diseases have variable clinical and pathological features, but are underlaid by the aggregation of misfolded proteins with a cytotoxic effect. Notably, abnormal activation of glial cells could mediate neuroinflammation, leading to the neurodegenerative condition. Microglia, a type of glial cell, a resident immune cell, form the forefront defense of the CNS immune system. Dysfunctional microglia and astrocyte, a different kind of glial cell with homeostatic function, impairs the protein aggregate (amyloid-beta plaque) clearance in AD. Studies have shown that microglia and astrocytes undergo alterations in their genetic profile, cellular and molecular responses, and thus promote dysfunctional immune cross-talk in AD. Hence, targeting microglia and astrocytes-driven molecular pathways could resolve the particular layers of neuroinflammation and set a reliable therapeutic intervention in AD progression.

## Introduction

Alzheimer's disease is a neurodegenerative disorder characterized by cognitive deficit and memory loss. The pathological characteristic of the disease is the extracellular accumulation of amyloid-beta (Aβ) plaque and intracellular neurofibrillary tangles (NFTs). NFTs are composed of the hyperphosphorylated microtubule-binding protein tau, which regulates the cytoskeleton range. Aβ plaque constitutes Aβ oligomer and fibrils generated by the catalytic cleavage of amyloid precursor protein (APP) by the enzyme gamma (γ) and beta (β)-secretase. Mutation of APP and γ secretase cause rare familial AD, emphasizing that Aβ may contribute to AD pathogenesis [1, 2]. The pathogenic role of Aβ is known, but the mechanism of pathogenesis remains unknown. Aβ aggregates affect neuronal development along with the cellular and molecular components of the immune system. Glial cells, including microglia and astrocytes, are known to propagate Aβ toxicity. Microglia acts as a sentinel that surveys and senses the changes within the brain. Aβ aggregates induce microglial activation, leading to the release of nitric oxide (NO), reactive oxygen species (ROS), pro-inflammatory cytokines, chemokine, which may contribute to neuronal death [3, 4]. Aβ aggregates engage with cellular receptors such as Toll-like receptors (TLRs) and receptors for advanced glycoxidation end-products (RAGE) on microglia. This engagement induces the transcriptional activation of downstream inflammatory response genes [5–7]. The binding of Aβ aggregates to TLRs and RAGE have also recognized on astrocytes. The activation of astrocytes referred as reactive astrocytes produces toxic factors due to the induction of downstream target genes [8]. The inflammation becomes detrimental when inflammatory responses establish feed-forward loops with the Aβ formation, which overwhelms the standard resolution mechanism of Aβ aggregates [3]. Moreover, the runaway inflammatory response imbalances the immune cross-talk between microglia, astrocytes, and neurons, affecting regulatory mechanisms of synaptic plasticity, neuronal survival, and cognition [9].

Furthermore, in contrast to a rare cause of familial AD, the altered expression of microglia-associated genes may promote the late onset of AD (LOAD) [10]. An allelic variant of the apolipoprotein E (APOE) gene is significantly associated with an increased risk of AD [11]. Microglia and astrocytes encoded APOE4 display an immunomodulatory effect; therefore, they actively participate in neuroinflammation. Besides APOE4, several other gene variants expressed on microglia such as Triggering Receptor Expressed on Myeloid Cells (TREM2), PLCG2, CD33, transient receptor potential melastatin 2 (TRPM2), etc., linked with increased risk of AD (10). Although genetic, biochemical studies have shown a potential role of neuroinflammation in AD pathogenesis, but are not solely considered the causative factor. However, there is always a pursuing question of whether resolving neuroinflammation would regress the AD progression. Thus, to effectively address this question, it will be vital to understand the role of microglia and astrocytes and the causative mechanism in AD pathology. Here, we review the microglia and astrocytes-mediated neuroinflammation, therapeutic interventions, and further prospects of neuroinflammation biology in AD pathology.

## The role of microglia in neuroinflammation

**Fig. 1:**
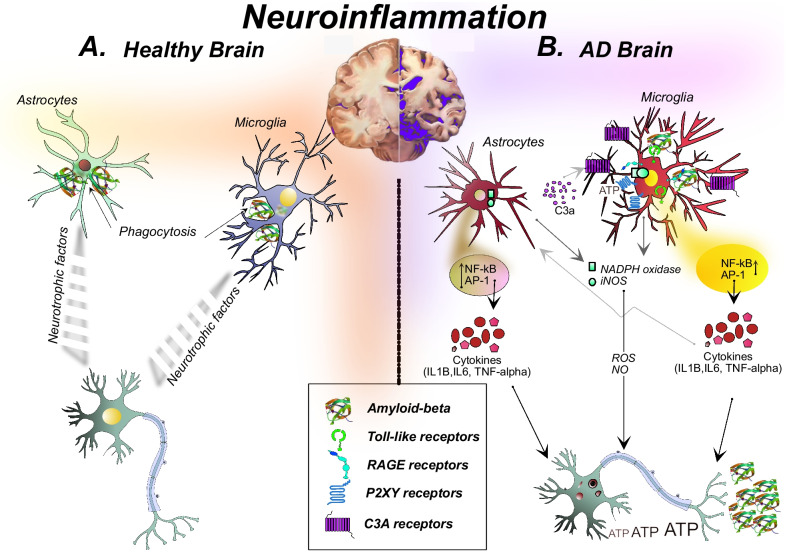
Schematic showing the modes of neuroinflammation in healthy and AD brain.** A** The healthy brain has minimal Aβ aggregates. Under normal physiological function microglia and astrocytes maintain neuronal homeostasis by clearing Aβ aggregates and providing neurotrophic factor to the brain. **B** The Alzheimer’s brain is associated with a large number of Aβ aggregates. The inhibited phagocytosis of Aβ aggregates and abrupt inflammatory response by microglia and astrocytes lead to the Aβ aggregates accumulation. Aβ aggregates bind to the pattern recognition receptors (PRRs) of microglia and stimulate downstream target genes NF-κB and AP-1. Subsequently, activated microglia produces cytokines. Cytokines contribute to astrocytes activation (referred to as reactive astrocytes) and affect neuronal health by causing neurotoxicity. The binding of Aβ aggregates to the microglia induces the NADPH oxidase and inducible nitric oxide synthase to produce ROS and NO leading to neurotoxicity. Likewise, Aβ aggregates bind to astrocyte receptors leading to the activation of downstream target genes NF-κB and AP-1 that subsequently produce cytokines. Cytokines affect neuronal health and cause neurotoxicity. The Aβ aggregates induce NADPH oxidase and inducible nitric oxide to produce ROS and NO by reactive astrocytes. The abrupt cross-talk between neurons, astrocytes, and microglia involving inflammatory molecules shown in the picture causes an imbalance in brain homeostasis and promotes neuronal death

Microglia are the brain resident macrophages derived from monocyte precursor cells during neurodevelopment. The two significant functional aspects of microglia are immune defense and homeostasis maintenance. Besides, they have an essential role in neurogenesis, the formation of the neuronal circuit, and maintaining neuron health [12–14]. Microglia undergo dynamic changes during the physiological and pathological conditions, exemplified by a change in its morphology from ramified (resting) to amoeboid (active) form [15]. Under normal physiological conditions, the microglia patrol the brain's microenvironment to provide immune surveillance and neuronal survival (Fig. 1A). Studies have shown that Aβ aggregates induce microglia to acquire a full spectrum of activation by acquiring morphological and molecular changes. This activated form of microglia is referred to as disease-associated microglia (DAM) and has overexpression of particular receptors, chemokines, cytokines, and other factors [16]. The pattern recognition receptors (PRRs) such as TLRs, RAGE, and nucleotide-binding oligomerization domain (NOD)-like receptors (NLRs) detect Aβ in the vicinity. The binding of Aβ, specifically to TLR4, has been suggested to activate microglia. Studies in the mouse model of AD have shown the role of TLR4 microglia signaling in mediating the increase of inflammatory cytokines production, phagocytosis inhibition, and higher plaque accumulation [17, 18]. Likewise, the binding of Aβ to RAGE promotes microglia activation-mediated neurotoxicity [7]. Besides, Aβ peptide activates NLRs, specifically the NLRP3, which triggers an inflammatory response by facilitating inflammasome complex formation and maturation of IL-1β [19]. Microglia express different purinergic receptors (e.g., P2Y12, P2Y6) that binds to extracellular nucleotides released by damaged cells. In turn, activated microglia have enhanced phagocytic potential [20, 21]. The PRRs ligation activates multiple transcription factors, such as NF-kB, AP-1, cAMP response element-binding protein, and interferon regulator families. These factors work in a combinatorial manner to regulate the expression of numerous downstream inflammatory response genes [22] (Fig. 1B).

Clinical and preclinical studies have shown the prominent role of activated microglia in AD progression [23]. Researchers have found microglia associated with Aβ plaque in AD patient's brains [3, 24, 25]. Microglia surrounding Aβ plaque has shown high immunoreactivity toward activation markers (e.g., MHCII and COX2), cytokines (e.g., IL-1, MCP-1, MIP-1α, IL-1β, TNFα, and IL-6), and chemokine receptors (e.g., CCR3, CCR5) [3]. MCP-1 induced chemotaxis of astrocytes and recruitment around Aβ plaque is reported [26]. Moreover, Aβ-induced microglia activation produces ROS through the NADPH oxidase system and reactive nitrogen species (RNS) by induction of inducible nitric oxide synthase (iNOS). These toxic factors stimulate amyloidogenesis, inhibition of Aβ clearance, and neurotoxicity [27] (Fig. 1B). In contrast, few studies have shown a neuroprotective effect of activated microglia. The research study has shown that the overexpression of IL-1β in the APP/PS1 mouse model reduces a load of Aβ plaque by increasing the number of activated microglia [28]. Another study has shown the benefit of IL-6 overexpression in APP transgenic mice against Aβ plaque accumulation by inducing gliosis and microglial activation [29]. Therefore, a discrepancy between studies suggests that microglia-mediated inflammation could be neurotoxic or neuroprotective. This may depend on the timing, duration, and amplitude of microglia activation.

## The role of astrocytes in neuroinflammation

Astrocyte is the most abundant glial cell of the CNS, derived from neural stem cells (NSCs) [30] (Fig. 1A). In the coarse similarity with microglia, astrocytes maintain homeostasis and provide metabolite and growth factors to neurons. Besides, astrocyte has an imperial role in synapse formation, synaptic plasticity, and modulates the extracellular balance of ions, fluid, removal of free radicals at the blood–brain barrier functions [31]. Astrocytes are an essential component of tripartite synapses where along with the inter-neuronal communications, bidirectional communication exists between neurons and astrocytes, which maintains homeostasis and neuronal survival [32]. Under pathological conditions, astrocytes undergo morphological and functional changes characterized by cell hypertrophy and excessive release of neurotoxic factors, referred to as reactive astrocytes. The reactive astrocytes have an increased expression of prominent markers such as glial fibrillary acidic protein (GFAP), and vimentin which underlies cell hypertrophy. In the context of AD, evidence from post-mortem and rodent model studies has shown the presence of reactive astrocytes in proximity with Aβ plaque [33, 34]. The depletion of GFAP and vimentin reduces the astrocytic reaction and increases the Aβ plaque load in the APP/PS1 mouse model of AD [35]. Reactive astrocytes may have a neuroprotective role. It surrounds (forms a physical barrier called glial scar) and infiltrates Aβ plaque to reduce collateral damage due to neurotoxic Aβ species. Also, in vitro and in situ studies have shown that reactive astrocytes clear or reduce Aβ deposits by phagocytosis [26, 36]. Moreover, by improving the phagocytic potential of astrocytes, impairment in a neural circuit, inflammatory response, and Aβ-mediated pathology could be restored, as suggested by the study [37].

However, several studies have shown the neurotoxic role of reactive astrocytes. Similar to microglia, astrocytes also sense Aβ aggregates in the TLR/RAGE-dependent manner, which leads to activation of downstream target genes and subsequent production of factors [8]. Aβ induces the increased production of neurotoxic factors, including ROS, NO, cytokines (e.g., IL-1β, IL-6, TNF-α, IFN-α, granulocyte–macrophage colony-stimulating factor, chemokines (e.g., MCP-1, MIP1-α, CCL4, IL-8, IFN-γ-inducible protein-10) [3, 38]. The astrocytes’ reaction has shown to be carried by four signaling pathways including, the Janus kinase (JAK)/STAT3, the calcium/calcineurin (CN)/nuclear factor of activated T-cells (NFAT), nuclear factor kappa-B (NF-κB), and the MAPK pathway [39] (Fig. 1B). Excessive production of neurotoxic factors modulates astrocytes APP processing homeostasis, which leads to increased Aβ load and toxicity. A study has shown that the treatment of TNF-α and IFN-γ or Aβ42 oligomers and fibrils to cultured primary astrocytes increases β-secretase processing of APP and Aβ load in AD transgenic mice. The study has revealed a significant role of the feed-forward loop driven by cytokines and Aβ42 in enhancing Aβ load [40]. Also, the contribution of inflammatory mediators to synaptic degeneration, cognitive impairment, and neuronal death has been reported [41, 42]. Particularly IL-1-mediated signaling is recognized for its pathogenic role in AD [43]. The study has shown abrogation of IL-1 signaling ameliorates impaired inflammatory response, improves cognitive deficit, reduces tau and Aβ pathology in a transgenic mouse model of AD [44]. Thus, existing evidence has shown the imperial role of astrocytes in neuroinflammation. Ongoing research studies are focusing on astrocytes-mediated neuroinflammatory pathways to resolve AD progression.

## Dysfunctional immune cross-talk cause neuroinflammation

Communication between immune cells and neurons is an essential process in CNS. The cross-talk between microglia, astrocyte, and neurons is necessary to provide homeostasis and neuronal survival. Dysregulated cross-talk is known to underlay neuroinflammation in AD. Soluble factors such as cytokines, chemokines, ATP, complement proteins, and growth factors are the players of immune cross-talk.

Cellular and animal studies have shown that the impaired activation of astrocytes alters microglia status in AD. Impaired astrocytes induce an increase in the number of microglia around Aβ plaque in GFAP − / − Vim − / − APP/PS1 mice which can be a compensatory mechanism to resolve the inefficient role of astrocytes as suggested by the study [34]. In the Aβ-induced mixed glial culture (microglia and astrocyte), the loss of the neurotoxic potential of microglia-conditioned media is because astrocytes modulate microglia activation, as shown by study [45]. Also, the inhibition of the CN/NFAT signaling pathway in activated astrocytes showed to modulate microglial activation and Aβ load in APP/PS1 AD mouse model [42]. Moreover, studies have reported the impact of complement proteins on the Aβ dynamics and microglia status in AD. The Aβ produced by neurons activates NFκB-signaling in astrocytes, which in turn releases complement protein C3a extracellularly. The C3a interacts with the C3a receptor (C3aR) on microglia and neurons and aggravates Aβ pathology by facilitating the feed-forward loop. The blocking of this pathogenic cycle by employing a C3aR antagonist reduces microglial activation and Aβ load. Thus, NFκB/C3/C3aR signaling has been shown to mediate the Aβ response by involving astrocytes and microglia [46, 47] (Fig. 1B). Besides, transforming growth factor-beta (TGF-β)-mediated modulation in microglia has been reported [48]. Impaired TGF-β signaling in the AD brain is shown [47, 49]. Also, the extracellular ATP release from N-methyl-D-aspartate (NMDA) induced stimulated neurons and astrocytes activates microglia by binding to purinergic receptors [50, 51]. And, there are many more factors by which astrocytes regulate microglia function, but the underlying mechanism is poorly understood.

Furthermore, there are reports which have shown that microglia could alter the astrocyte status in CNS. The activated microglia induce A1 astrocyte, a subtype of reactive astrocyte, by secreting cytokines IL-1α, TNF, and C1q. A1 astrocytes are neurotoxic (Fig. 1B). The presence of A1 astrocyte in human AD post-mortem tissue is reported [52]. The research studies have highlighted the beneficial effect of neuropeptide in reducing neuroinflammation in AD. Recent study has shown that glucagon-like peptide-1, a gut-derived incretin agonist has reduced Aβ-induced microglia activation. In turn, executes the formation of reactive astrocytes concomitantly leading to inhibition of neuroinflammation in AD model [53]. Thus, evidence implies that astrocytes and microglia regulation are interdependent. The impairment in existing intercommunication may lead to neurotoxicity.

## Role of cellular mechanisms in the regulation of microglial-mediated neuroinflammation

Studies have focussed on the biosynthetic mechanisms, derived metabolites, and mediators regulating microglia in AD pathogenesis. The sphingosine kinase1 (SphK1), an ATP-dependent lipid kinase, regulates microglia. SphK1 causes the catalytic reaction of acetyl COA and sphingosine to generate N-acetyl sphingosine (N-AS). N-AS acetylates cyclo-oxygenase 2(COX 2) and increases pro-resolving mediators (SPM). The treatment of NAS in APP/PS1 mice has shown to increase SPM, which resolves neuroinflammation by upregulation of microglia genes linked with phagocytosis in comparison to untreated APP/PS1 mice. In addition, Aβ-treated microglia showed less N-AS while treatment of N-AS has increased the production of SPM, consequently resolving neuroinflammation [54]. Studies have shown a key role of COX/prostaglandin E 2(PGE2) in the pathogenesis of AD. Genetic deletion of the PGE2 EP2 receptor on microglia has shown beneficial effects by enhancing phagocytosis of Aβ, resolving neuroinflammation, maintaining trophic factors, and signalling. Moreover, improvement in cognitive behavior and synaptic proteins in the mice model of AD [55, 56]. Neuro-inflammation has been associated with disturbed metabolic processes leading to over-production of reactive oxygen species. NADPH oxidase is a multi-subunit enzyme complex that drives the production of reactive oxygen species, and its role in microglia-mediated neurotoxicity is shown [57, 58]. Microglia exhibits classical M1 and alternative M2 phenotypes in pathological conditions. M1 phenotype facilitates the production of inflammatory cytokines leading to a neurotoxic effect, while M2 produces anti-inflammatory cytokines. The study has shown that deletion of NADPH oxidase causes microglia to switch from M1 to M2 phenotype, thus protecting neurons against neuroinflammation [59]. The alteration of the microglia state from M2 to M2 has shown a beneficial effect in Alzheimer’s model. Research studies have found induction of the M2 microglia state ameliorates Aβ-induced toxicity and improves cognition in Alzheimer’s disease model [60]. The advent of single-cell RNA sequencing/ScRNA and single nucleus sequencing/snRNA has enabled researchers to identify the heterogeneity of microglia subpopulations under normal and pathological conditions. ScRNA/snRNA datasets have paved the way to develop a therapeutic strategy against DAM and resolve neuroinflammation [61]. Researchers are focussing on the implication of microglia deletion in a pathological context. Studies have found that upon microglia deletion the niche is repopulated by microglia that come from differentiated microglia progenitor cells, residual microglia, or infiltrating peripheral cells [62–64]. In the context of AD, the beneficial effect of microglia depletion has shown by the administration of a colony-stimulating factor 1 receptor (CSF1 R) inhibitor in the mice model of AD. CSF1R is an essential factor for maintaining microglia health. The inhibition of microglial CSF1R has improved cognition [65], rescued dendritic formation, resolved neuroinflammation [66], and reduced amyloid-beta plaque formation [67]. Notably, depletion of microglia followed by repopulation of newborn microglia has opened the way to explore therapeutic approaches in a pathological context. Taken together, microglia modulation is a tantalizing attempt to modulate neuroinflammation-mediated neurotoxicity.

### Calcium and P2XY signaling in regulating microglia-mediated inflammation

Calcium homeostasis plays a crucial role in microglia activation. Emerging studies have found a role of calcium homeostasis modulators (Calhms) in regulating calcium homeostasis in neurological disorders. Calhms are the voltage-gated calcium ion channel that allows the permeability of ions and ATP in a voltage-dependent manner. It gets activated upon the abrupt extracellular concentration of calcium and plays a significant role in controlling neuronal excitability, taste signalling, and pathologies of depression and AD. Genetic study has identified that CALHM1 P86L polymorphism is associated with incidence of AD. The role of calhm1 in regulating calcium homeostasis, Aβ production, and neuronal cell viability against Aβ-induced neurotoxicity has been shown [68]. The increased level of calhm2 has been noticed in AD pathogenesis. Moreover, the dissection of the mechanistic pathway reveals that calhm2 regulates calcium homeostasis, and inflammation in the microglia mice model of AD [69]. Calhm2 regulates the functional interaction of purinergic receptor P2XY (a cation channel expressed highly on microglia) with NLRP3. Inhibition of P2XY has reduced inflammation and increased the phagocytic potential of microglia [70], which in turn provides a beneficial effect on AD pathogenesis [71, 72]. In the study, the knockout of calhm2 decreases P2XY leading to a reduction in its interaction with NLRP3, consequently reducing neuroinflammation [69]. Research lines have identified the protein structure of calhm2 [73, 74], which has paved the way for understanding and developing therapeutic strategies against neurological diseases [Table 1].

**Table 1:** Role of cellular mechanisms in the regulation of microglia-mediated neuroinflammation

Mechanism	Mediator	Effects	Significance in AD	References
SphK1	N-AS	Acetylates COX and increases SPM SPM upregulates microglial phagocytic potential	Resolves neuroinflammation Reduces Aβ aggregates	[54]
COX/PGE2	PGE2 EP2 receptor of microglia	Deletion of PGE2 EP2 on microglia: Enhances phagocytosis of Aβ	Reduces neuroinflammation Reduces Aβ aggregates Maintain trophic factor and signalling	[55, 56]
NADPH oxidase-mediated metabolic pathway	Enzyme NADPH oxidase	Deletion of NADPH oxidase: Reduces ROS production Allows microglial switch from M1 to M2 phenotype	Reduces oxidative stress Decreases neuroinflammation	[57, 58]
CSFIR-mediated signalling	CSFIR	Deletion of CSFIR of microglia: Decreases microglia in niche Repopulation of new born microglia	Improves cognition Reduces Aβ burden Rescue dendrites Resolve neuroinflammation	[66, 67]
Calhms	Calhm1 Calhm2	Calcium homeostasis Aβ production Neuronal cell Viability Calcium homeostasis Neuro-inflammation	CALHM1 P86L polymorphism is associated with the incidence of AD Ablation of Cahm2 inhibits the production of inflammatory cytokines	[68] [69]
P2XY-NLRP3 pathway	PRXY	Regulates production of inflammatory cytokines via interaction with NLRP3	Inhibition of P2XY reduces neuroinflammation	[69,70,71, 72,73,74]

## Microglia expressed gene variants associated with AD

Genetic studies have elucidated different immunomodulatory gene variants associated with AD pathogenesis. Mutation in APP and γ-secretase components presenilin 1 and 2 causes rare familial AD [75]. Gene variants expressed on microglia have shown an association with the development of LOAD. The most studied and established risk factor is allele E4 of the apolipoprotein E gene. And, gene variants of TREM2, phosphoinositide phospholipase Cγ2 (PLCG2), CD33, TRPM2 are associated with LOAD [76, 77].

## The role of APOE in microglial regulation

Human genetics studies have elucidated the majority of genes expressed on microglia and associated with AD risk [9]. Transcriptomics studies have suggested microglia acquire alteration in gene expression profile during the transition from homeostatic phenotype to molecular phenotype called DAM phenotype [78–80]. Genome-wide association studies (GWAS), cellular and animal model studies have shown a strong association of apolipoprotein E with the high risk of AD. Strittmatter and Roses were the first to suggest the role of APOE in AD [81], were further supported by other studies. The APOE plays physiological functions within the brain, but a significant role is to transport cholesterol, high-density lipoprotein particles. In humans, three polymorphic forms of APOE are present such as APOE2, APOE3, and APOE4. APOE4 is strongly associated with a high risk of AD. Numerous studies emphasize that the APOE4 modulates microglia function and contributes to neurotoxicity. A human cellular model study has shown the isogenic conversion of human iPSC-derived microglia from APOE3/E3 AD patients to APOE4/E4. Transitioned APOE4 microglia was found transcriptionally similar to APOE4 of the human AD brain. The study has configured that in human AD DAM phenotype of microglia might be induced by APOE4 [82]. APOE and its receptor have crucial role in AD. Studies have suggested that APOE4 is less effective in clearing Aβ aggregates because it has less tendency to bind Aβ peptides in comparison to APOE3 [83]. Noticeably, the APOE4 regulates the aggregation and clearance of Aβ aggregates and contributes to Aβ plaque accumulation [84]. The APOE4-mediated increase in the levels of pro-inflammatory cytokines (e.g., TNF-α, IL-6, and IL12p40) more efficiently than APOE3 has shown [85]. The human clinical study data obtained from two Chinese populations corroborate the above finding where APOE4 carriers had an increased level of pro-inflammatory (TNF-α, IL-6, and IL-1β) cytokines than the carriers of APOE2 and APOE3 [86]. Thus, APOE4 plays a crucial role in AD pathology and could be a target for therapeutic intervention.

## The role of TREM2 in microglia regulation

GWAS studies have identified some rare gene variants expressed on microglia which contribute to AD pathology. In 2013, two independent studies from distinct populations deciphered the whole-exome and whole-genome sequencing and showed that R47H options of TREM2 had an increased risk of developing AD [87, 88]. TREM2 is a microglia cell surface receptor that provides several physiological functions to microglia. TREM2 stimulation (triggered by tyrosine phosphorylation) promotes microglial chemotaxis, phagocytosis, proliferation, and survival [89–92]. TREM2 has reported to regulate microbial function by maintaining the cellular energetics and biosynthetic metabolism [93]. The common extracellular ligands of TREM2 are anionic lipid, APOE, Aβ, and apoptotic cells [94, 95]. Impairment in TREM2–ligand interactions have been noticed in TREM2 variants, which can be a causation factor promoting the increased risk of AD [94, 96]. Aβ aggregates bind efficiently to lipoproteins, which in interaction with TREM2 microglia triggers downstream activation of target genes that eventually mediate Aβ clearance. However, TREM2-deficient microglia have reduced uptake of Aβ-lipoprotein complexes [94, 97]. Also, the TREM2 deficiency have shown higher Aβ plaque accumulation and dysfunctional microglia in mouse model of AD [95, 98]. The supraphysiological expression of TREM2 has shown improvement in microglia function, and amelioration in neuropathological and behavioral deficit in AD mouse models [99]. Also, a study has shown that TREM2 facilitates microglia-mediated phagocytosis of APOE bounded apoptotic neurons [100]. Thus, TREM2 could be an important regulatory factor of microglia, as its impairment may lead to Aβ pathology.

## The role of TRPM2 channel in microglia regulation

Recently, the research line has focused on a receptor TRPM2 which is majorly expressed on microglia and has a role in microglia regulation. The TRPM2 channel is a ligand-gated calcium-permeable cation channel, co-activated by intracellular ADP ribose (ADPR) and calcium and has shown high sensitivity toward oxidative stress or ROS [101–104]. TRPM2 channel has critical physiological functions such as body temperature regulation, immune response, and apoptosis [105–108]. In 2004, Kraft et al. were the first to examine the expression of the TRPM2 channel in rodent’s microglial cells. The research has revealed novel calcium influx/signaling pathways mediated by the TRPM2 channel upon activation by H_2_O_2_ and ADPR [109]. And it was supported by the study, which has shown oxidative stress-induced calcium influx through the TRPM2 channel in human cultured microglia [110]. Moreover, the genetic deletion of TRPM2 showed to reverse the Aβ-induced perturbations such as microglial activation, neuronal toxicity, and memory impairment in APP/PS1 mice [111]. Continuingly, the research line has revealed the molecular mechanism of Aβ-induced activation of TRMP2 in cultured mouse microglia. Aβ-induced ROS (produced in protein kinase C/NADPH oxidase-dependent manner) activates poly (ADPR) polymerase‐1 (PARP-1), which acts on NAD to generate ADPR. Subsequently, the ADPR induces the activation of the TRPM2 channel. Also, TRPM2 channel-mediated activation of PYK2/MEK/ERK signaling acts as a fuel for PARP-1 and TRPM2 signaling. This leads to the sustained activation of TRPM2 channel with cytotoxic affects [112]. Thus, based on considerable evidence, TRPM2 can be regarded as a new player in neuroinflammation and could be used as a therapeutic intervention against AD.

## Genetic variants linked with astrocytes

Few genes expressed on astrocytes have an immunomodulatory effect and undergo altered expression in AD [113]. The APOE gene variant APOE4 has shown an impact on Aβ species degradation and clearance. Here** w**e discuss the role of astrocytes APOE gene variants in AD pathogenesis.

## The role of APOE in astrocyte regulation

Inconsistent with microglia, astrocytes APOE gene variants too associate with neuroprotective or neurotoxic effects in AD by regulating Aβ dynamics. The study has shown that the APOE-deficient mouse astrocytes were inefficient in clearing Aβ plaque compared to wild-type mouse astrocytes in vitro [114]. The cell culture, animal model, and human study have deciphered a strong association of astrocytes APOE4 with AD pathogenesis. But the underlying mechanism is poorly understood [115–118]. The APOE4 astrocytes showed to inhibit Aβ clearance and promote Aβ accumulation. It affects initial amyloid plaque development [115]. In vivo study has highlighted a mechanistic view of APOE4 astrocytes-mediated impairment in Aβ clearance. APOE4 astrocytes induce the acidification of endosomal pH by downregulating Na + /H + exchanger isoform NHE6. It is a critical leak pathway for endosomal protons. It has led to the intracellular sequestration of low-density lipoprotein receptor-related protein (LRP1), leading to the loss of Aβ clearance. Epigenetic modifiers have restored NHE6 expression, normalized APOE4-specific defects in endosome pH, LRP1 trafficking, and Aβ clearance. The study has proposed endosomal pH as a target for the correction of amyloid disorders [119]. Besides, several studies have shown a higher contribution of APOE4 in tau pathogenesis and neuroinflammation relative to other APOE isoforms. Research study has shown APOE4 exerts neurotoxicity by influencing tau pathology and neuroinflammation. However, the absence of APOE was protective [120]. Taken together, APOE performs a complex set of interrelated functions in astrocytes, and impaired APOE expression affects neuronal survival.

## Connecting link between neuroinflammation and autophagy

Autophagy is a catabolic process that maintains intracellular homeostasis by clearing misfolded protein and damaged organelles. Autophagy impairment has been associated with several neurodegenerative diseases like AD. The autophagy process involves a series of crucial steps responsible for delivering damaged organelles or protein aggregates to lysosomal degradation. The accumulated autophagic vacuoles (AVs) within dystrophic neurites indicate incomplete autophagy in AD [121] Studies have shown connecting link between the accumulation of Aβ aggregates and dysfunctional autophagy [122–125]. Emerging evidence has found the existing relation between neuroinflammation and autophagy. In vitro study has shown that the IL-1beta cytokine induces autophagy indicated by the accumulation of autophagosomes loaded with p62 and LC3 in triculture and pure culture of microglial [126]. Another study has found that IL-1 beta reduces inflammation and enhances microglial phagocytosis of Aβ, providing a beneficial effect in the 3xTgAD model [127]. Autophagy plays a role in facilitating the secretion of IL-1beta [128]. The anti-inflammatory role of autophagy has come into consideration due to its role in scavenging mitochondrial ROS and suppressing NLRP3 inflammasome activation [129, 130]. Moreover, defect in autophagy protein has been associated with microglia-mediated neuroinflammation. The study has shown the defect in microglial beclin 1, an essential protein for induction of autophagy, mediates neuroinflammation [131]. Researchers are interested in exploring the therapeutic use of autophagy enhancers against neurodegenerative diseases by employing animal models. The translation of preclinical outcomes could be different when implicated for clinical use because of species variation. The scientific community is utilizing human-induced pluripotent stem cells (hi-PSCs) that have paved the way to mirror the effect of therapeutic strategies in vivo. The majority of cases of AD are sporadic, few are familial, and modeling sporadic AD in rodents does not exist. The hi-PSCs neuronal model for sporadic AD has paved the way for exploring the therapeutic strategies encompassing autophagy modulators [132]. Studies have suggested diverse ways of autophagy enhancement that could provide potential benefits concerning neurodegenerative diseases. The autophagy inducer rapamycin, an inhibitor of mTOR, has shown a beneficial effect in several cellular and animal models of AD [133–135].

Rapamycin along with other compounds has shown enhancement in autophagy. The combinatorial effect of autophagy modulators has been suggested to provide better output in AD pathogenesis [136]. It can be hypothesized that autophagy enhancers could reduce neuroinflammation, in turn, protects neurons against the toxicity of protein aggregates in neurodegenerative conditions like AD.

## Therapeutic approaches to combat neuroinflammation in AD

Considerably, neuroinflammation occurs before the appearance of histopathological or pathological features in AD. Therefore, existing as well as ongoing therapeutic interventions rely on the asymptomatic and prodromal stages of AD. Herbal medicines have shown beneficial effects in AD pathogenesis, probably by dampening neuroinflammation.

Curcumin and its metabolite tetrahydrocurcumin a derived from the spice turmeric, has shown a neuroprotective effect in cell culture and animal model studies of AD [137–140]. Curcumin-mediated beneficial effects such as modulation in inflammatory responses, reduction in Aβ plaque accumulation, protection against synaptic toxicity, and improvement in cognitive function enforce the use of curcumin in clinical trials for treating AD. A review of Voulgaropoulou et al., 2019 has summarized preclinical and clinical data encompassing the curcumin effect on cognitive impairment in AD and normal aging. Curcumin has a cognition enhancer property shown in AD and non-pathological aging [141]. Moreover, quercetin flavonoid derived from mulberry fruit Morus alba is rich in phenolics, vitamin c, linoleic acid, etc., and possesses anti-inflammatory and anti-oxidant properties [142]. Quercetin inhibits the NF-kappa B pathway and upregulates the anti-oxidant defense system NRf2/HO-1 axis [143].

Resveratrol, a polyphenol, commonly present in grapevine and other fruits, has shown beneficial effects in AD. Studies suggest that resveratrol has anti-oxidant, anti-inflammatory, and neuroprotective properties. The anti-oxidant and anti-inflammatory effect of resveratrol is due to the activation of Silent Information Regulator-1 [144]. Treatment of resveratrol in AD patients has shown amelioration in inflammatory responses, a decrease in Aβ levels, and improved cognitive function [145]. Furthermore, terpenoids such as *Ginkgo biloba* have shown neuroprotection in neurodegenerative conditions like AD. *Ginkgo biloba* has anti-inflammatory, anti-oxidant, and anti-apoptotic effects in the neurological diseases model [146]. It increases SIRT-1 expression, inhibits NF-kB, upregulates heme oxygenase-1 (HO-1), anti-apoptotic protein expression, and down-regulates pro-apoptotic protein expression [147]. It ameliorates mitochondrial dysfunction and protects SH-SY5Y cells against the toxic incidence of ROS [148]. In Aβ25–35-induced cultured hippocampal neurons, *Ginkgo biloba* has upregulated brain-derived neurotrophic factors and inhibited apoptosis [149]. Studies have also shed light on the compound Sinomenine, an alkaloid, extracted from the Chinese medicinal plant, Sinomenium acutum. In China, Sinomenine is a recommended medicine for the patient against rheumatoid arthritis. It has shown neuroprotection against Aβ-induced glial-mediated toxicity to neurons [150, 151]. The mechanism of Sinomenine is not clearly understood. The study by Qian and colleagues has shown that Sinomenine inhibits NADPH oxidase and protects neurons against the toxic effect of lipopolysaccharide (LPS) and the 1-methyl-4 phenylpyridinium (MPP +)- in a model of PD [152]. The dissection of the molecular mechanism of Sinomenine will be interesting and could uncover the therapeutic targets for AD. Many more natural compounds have shown potential efficacy toward AD, but have not included because this article has other orientations too.

Besides natural compounds, several synthetic drugs are in clinical research of AD and have modulated inflammatory responses. Drugs targeting cell surface receptors (TLRs, RAGE) or transducers NF-κB/effector (TNF-α) of the innate immune system are in the research.

GC021109 binds a purinergic receptor (P2Y6) expressed on microglia. It stimulates microglial phagocytosis and inhibits the release of inflammatory cytokines. GC021109 is in the early phase of a clinical trial [153]. Secondly, Azeliragon, an antagonist of RAGE, blocks the interaction of Aβ − RAGE and has shown neuroprotection in AD transgenic mouse models [154]. Pioglitazone is an agonist of peroxisome-proliferator-activated receptor gamma (PPARγ), which regulates astrocyte/neuron metabolic coupling, dendritic formation, and synapses. Pioglitazone has shown beneficial effects against several pathological features of AD including disturbed bioenergetic, inflammation, oxidative stress, microglial defects, etc. It improves learning and memory, synaptic activity, and amyloid and tau pathology [155]. Despite promising preclinical data, the drug failed in the clinical trial due to a lack of efficacy.

Besides, epidemiological studies have shown that the use of nonsteroidal anti-inflammatory drugs (NSAIDs) reduces the risk of developing AD by modulating the inflammatory process [156, 157]. But most of the tested NSAIDs failed in AD clinical trials majorly due to a lack of efficacy in improving AD symptoms [158]. Researchers have now focused on a new approach to combat AD pathology. The combination therapy of ibuprofen and cromolyn (ALZT-OP1) is on a clinical trial to combat the early stages of AD [159, 160]. Few drugs regulating cytokine production and activity are under investigation. Neflamapimod, an inhibitor of the intracellular enzyme p38 MAPK α, has shown promising results in cellular and animal studies of AD. Neflamapimod showed a reduction in Aβ load, ameliorate synaptic function, and reverses memory deficits [159–161]. The drug has passed the phase 2a clinical trial with a positive outcome and forwarded for phase 2b clinical trial. Conclusively, preclinical data of anti-inflammatory medications are reliable. But for clinical use, they should be accessible to CNS and could target mediators of neuroinflammation [Table 2].

**Table 2:** Therapeutic approach to combat neuroinflammation in AD

Phytochemical origin	Essential components			Effects	Significance in AD		References	
*Natural products*								
Turmeric	Curcumin Tetrahydrocurcumin			Inhibits gliosis Reduces oxidized protein Disaggregates Aβ Prevent synaptic toxicity	Modulates inflammation Reduces Aβ aggregates Protects synapses Improves cognition		[137,138,139,140,141]	
*Morus alba*	Quercetin			Inhibits the NF-kB pathway Upregulates the anti-oxidant defense system NRf2/HO-1 axis Regulates apoptosis	Reduces neuroinflammation Reduces oxidative stress		[142, 143]	
Grapevine and other fruits	Resveratrol			Inhibits gliosis Upregulates SIRT-1 expression Reduces oxidative stress	Reduces neuroinflammation Decreases Aβ aggregates Improves cognition		[144, 145]	
*Ginkgo biloba*	Ginkgolides			Increases SIRT-1 expression Inhibits NF-kB pathway Upregulated HO-1 expression Upregulates anti-apoptotic protein expression	Reduces neuroinflammation Anti-apoptotic Protects neurons		[146–149]	
*Sinomenine acutum*	Sinomenine			Inhibits gliosis Inhibit NADPH oxidase Prevent DNA damage	Reduces neuroinflammation Reduces ROS production Protects neuron		[150–152]	
Name		Target	Effects			Significance in AD		References
Synthetic drugs:								
GC021109		P2XY	Enhances phagocytosis by microglia			Inhibits neuroinflammation		[153]
Azeliragon		Antagonist of RAGE	Prevent Aβ-induced neurotoxicity			Neuroprotection		[154]
Pioglitazone		Agonist of PPARγ	Ameliorates metabolism of neuron Reduces neuroinflammation Reduces oxidative stress			Improves learning and memory Improves synaptic activity Reduces Aβ and tau pathology		[155]
Ibuprofen		COX	Inhibits cytokine production			Reduces neuroinflammation		[159]
Cromolyn		Microglia	Inhibits release of inflammatory cytokines			Reduces neuroinflammation		[160]
Neflamapimod		Intracellular enzyme p38MAPKα	Inhibits inflammatory pathway			Improves cognition Ameliorates synaptic dysfunction		[161,162,163]

## Conclusion and future perspectives

Neuroinflammation is a central mechanism in the pathogenesis of AD. It consists of a complex network of interrelated processes involving roles played by microglia and astrocytes. Unbalanced activation of astrocytes and microglia instigates abnormal inflammatory responses leading to neuronal death. Also, a perturbation in existing cross-talk between microglia, astrocytes, and neurons causes neurotoxicity and cognitive deterioration. Moreover, genetic variants associated with astrocytes and microglia contribute to AD progression. Targeting genetic variants could be a promising therapeutic intervention but challenging too. Drugs are in the clinical research line, aiming to reduce AD progression or revert AD pathology by targeting neuroinflammation. Natural compounds have shown a minimum level of side effects with a promising outcome. Therefore, combination therapy involving natural compounds with other interventions could lead to a better therapeutic approach toward AD. Besides, ongoing recent studies target microglia and astrocyte activation state, trying to revert them into the homeostatic phenotype, which may lower the consequences of inflammatory responses. The existing link between inflammation and autophagy has attracted the attention of researchers towards improving autophagy defects. The available literature suggests that enhancing autophagy could prove to be a promising therapeutic strategy against the toxicity of protein aggregates in neurodegenerative conditions. Improving autophagy defects may ameliorate neuroinflammation and could be a therapeutic intervention toward AD progression.

## Data Availability

The author declares that the relevant data are included in the article.

## Abbreviations

CNS: Central nervous system
AD: Alzheimer’s disease
Aβ: Amyloid beta
NFTs: Neurofibrillary tangles
APP: Amyloid precursor protein
NO: Nitric oxide
ROS: Reactive oxygen species
TLRs: Toll-like receptors
RAGE: Receptors for advanced glycoxidation end-product
SphK1: Sphingosine Kinase 1
N-AS: N-Acetyl sphingosine
SPM: Pro resolving factor
PGE2: Prostaglandin E2
CSF1R: Colony stimulating factor 1-receptor
Calhms: Calcium homeostasis modulators
LOAD: Late onset of AD
TREM2: Triggering receptor expressed on myeloid cells
TRPM2: Transient receptor potential melastatin 2
DAM: Death-associated microglia
PRRs: Pattern recognition receptors
NOD: Nucleotide binding oligomerization domain
NLRs: NOD-like receptors
RNS: Reactive nitrogen species
iNOS: Inducible nitric oxide synthase
NSCs: Neural stem cells
GFAP: Glial fibrillar acidic protein
CN: Calcium/calcineurin
NFAT: Nuclear factor of activated T cells
NF-κB: Nuclear factor Kappa B
HO-1: Heme-oxygenase-1
C3aR: C3a receptor
TGF-β: Transforming growth factor
NMDA: N-Methyl-D-aspartate
GWAS: Genome-wide association studies
ADPR: ADP ribose
PARP-1: Ploy (ADPR) polymerase-1
LRP1: Lipoprotein receptor-related protein
NSAIDs: Nonsteroidal anti-inflammatory drugs
